# Meridian-Specific and Post-Optical Deficits of Spatial Vision in Human Astigmatism: Evidences From Psycho-Physical and EEG Scalings

**DOI:** 10.3389/fpsyg.2021.595536

**Published:** 2021-03-17

**Authors:** Li Gu, Yiyao Wang, Lei Feng, Saiqun Li, Mengwei Zhang, Qingqing Ye, Yijing Zhuang, Zhong-Lin Lu, Jinrong Li, Jin Yuan

**Affiliations:** ^1^State Key Laboratory of Ophthalmology, Zhongshan Ophthalmic Center, Sun Yat-sen University, Guangzhou, China; ^2^Division of Arts and Sciences, NYU Shanghai, Shanghai, China; ^3^Center for Neural Science, Department of Psychology, New York University, New York, NY, United States; ^4^NYU-ECNU Institute of Cognitive Neuroscience, NYU Shanghai, Shanghai, China

**Keywords:** contrast sensitivity, sVEP, astigmatism, meridional amblyopia, spatial vision

## Abstract

Previous studies have demonstrated that orientation-specific deprivation in early life can lead to neural deficits of spatial vision in certain space, and can even result in meridional amblyopia (MA). Individuals with astigmatism are the optimal and natural models for exploring this asymmetric development of spatial vision in the human visual system. This study aims to assess the contrast sensitivity function (CSF) and EEG signals along two principal meridians in participants with regular astigmatism when being optimal optical corrected. Twelve participants with astigmatism (AST group, 20 eyes) and thirteen participants with (MA group, 19 eyes) were recruited in the current study. CSFs and spatial sweep visual evoked potentials (sVEP) were measured with vertical and horizontal sinewave gratings along two principal meridians monocularly. Area under log CSF (AULCSF), spatial frequency threshold corresponding to 80% contrast gratings (SF threshold at 80% ctr), and CSF acuity were calculated from CSF test. In addition, sVEP amplitudes and thresholds were calculated with the recursive least square method. Participants with astigmatism exhibited marked vertical-horizontal resolution disparities even after they were corrected with optimal optical corrections. CSF tests showed that AULCSF along weak meridian (measured with horizontal gratings) was lower than that along strong meridian (measured with vertical gratings) in both groups. Significant meridional disparity of CSF acuity was also found in both groups. In addition, the MA group showed larger meridional disparity compared to the AST group. Spatial sVEP thresholds also supported the existence of marked meridional disparity. Our results suggest that meridian-specific partial deprivation in early life might lead to monocularly asymmetric development of spatial vision in the human visual system. In terms of application, we tested the feasibility and reliability of adopting psychophysical and EEG scalings to investigate the asymmetric development of spatial vision related to astigmatism. These paradigms are potentially applicable to reduce and even eliminate the meridional disparity in the primary visual cortex by adopting perceptual learning or other vision-related interventions.

## Introduction

The development of visual system is extraordinary sensitive to early visual experience (Berardi et al., 2000; Knudsen, 2004; Kiorpes, 2016). Abnormal visual experience will result in functional and structural deficits in the visual pathway and cerebral cortex, such as deprivation amblyopia (Kiorpes and McKee, 1999; Kiorpes, 2006). In the past decades, psychophysical, electrophysiological, and neuro-imaging techniques make the exploration of neural mechanism underlying human amblyopia feasible and fruitful. However, less attention has been given to meridional amblyopia (MA) resulting from astigmatism. Animal studies have consistently demonstrated that visual deprivation along one orientation would lead to orientation-specific deficits in the visual pathway and cerebral cortex (Blakemore and Cooper, 1970; Hirsch and Spinelli, 1970). However, systematic and comprehensive investigation of meridian-specific deficits in the human visual system is still lacking.

Individuals with astigmatism are the optimal model for investigating the asymmetric meridional development of spatial vision in the human neural system. Astigmatism is a common condition of refractive error in which the eye’s refractive power differs in various meridians, with maximum and minimum powers mutually perpendicular (Read et al., 2014). There are two categories of astigmatism, regular, and irregular astigmatism. The former is normally from abnormal development of corneal curvature, and the latter is from the influence of ocular disease on the components of optical media. Astigmatism has a high prevalence (over 25%) in eastern Asia population most probably due to the different anatomical structure between eyelid, orbit, and eyeball (He et al., 2004, 2007; Rim et al., 2016; Nakamura et al., 2018; Wang et al., 2019).

For regular astigmatism, the parallel rays of light entering the eye are brought to a focus at two distinct focal lines perpendicular to each other rather than to a single focal point (Read et al., 2014). The asymmetrical input of visual signal in perpendicular meridians will affect visual functions, and may even result in MA (Mitchell et al., 1973; Gwiazda et al., 1985). Patients with MA have substandard corrected visual acuity behaviorally, and more notably, astigmatic individuals and amblyopes showed the abnormal development of spatial vision in the visual system (Fiorentini and Maffei, 1973; Freeman and Thibos, 1973; Mitchell et al., 1973; Freeman, 1975). Freeman and his collaborators (Freeman et al., 1972) first demonstrated that astigmatism may contribute to meridian-specific neural deficits in spatial vision, since certain astigmatic participants showed substantial vertical-horizontal resolution differences, even when being fully corrected optically. Since then, researchers further reported on an extensive investigation of the meridional differences in resolution with contrast sensitivity and electrophysiological evidence (Fiorentini and Maffei, 1973; Mitchell et al., 1973; Freeman, 1975). These results indicated that meridian-specific partial deprivation may lead to an analogous modification in the organization of neurons in the human visual system (Freeman et al., 1972). Following previous studies, we used the meridional disparity to represent the resolution differences between vertical and horizontal meridians. However, psychophysical and electrophysiological evidence from a recent study (Yap et al., 2019) demonstrated that non-amblyopic children with and without astigmatism also showed meridional disparity.

Contrast sensitivity and visual evoked potentials (VEPs) have been widely used to investigate the influence of stimulus orientation on the spatial tuning function in healthy subjects and patients (Freeman and Thibos, 1973; Tobimatsu et al., 1993; Arakawa et al., 2000; Tobimatsu and Celesia, 2006; Yap et al., 2019, 2020a). The computerized contrast sensitivity function (CSF) paradigm assesses spatial vision over a wide range of spatial frequencies and contrast levels, and has been demonstrated to be a suitable and applicable paradigm for detecting and diagnosing deficits in spatial vision by a handful of studies (Zhou et al., 2006; Yenice et al., 2007; Huang et al., 2008). Spatial sweep visual evoked potentials (sVEP) has been adopted to measure spatial acuity (Regan, 1977; Tyler, 1979; Norcia and Tyler, 1985; Good and Hou, 2006; Hou et al., 2011; Norcia et al., 2015). The combination of both two scalings would furnish subjective and objective paradigm to future studies related to the improvement of spatial vision, such as diminishing the meridional disparity via perceptual learning in the future study.

Therefore, the goal of the present study was to verify the feasibility and reliability of combining psycho-physical and EEG scalings to build human model of asymmetrical spatial vision development in individuals with astigmatism. To this end, the computerized CSF and sVEP tests were employed. CSF paradigm was adopted to assess spatial vision over a wide range of spatial frequencies and contrast levels (Zhou et al., 2006; Yenice et al., 2007; Huang et al., 2008) and spatial sVEP was adopted to measure spatial acuity (Regan, 1977; Tyler, 1979; Norcia and Tyler, 1985; Good and Hou, 2006; Hou et al., 2011; Norcia et al., 2015). Here, the Area under log CSF (AULCSF), spatial frequency threshold corresponding to 80% contrast gratings (SF threshold at 80% ctr), and CSF acuity were calculated from CSF tests, and the sVEP thresholds were calculated to evaluate meridional disparity in spatial acuity. In these two different paradigms, grating stimuli were presented either along the vertical or the horizontal meridian. Notably, the meridional disparity was quantified under full optical correction with spectacles or contact lenses to eliminate the influence from optical errors. From this, we expect to explore the asymmetrical visual development in human astigmatism in a comprehensive and systematic way.

## Materials and Methods

### Participants

Twelve participants with astigmatism (AST group, 20 eyes, 6 males, mean age = 9.10 ± 1.37 years, age range:) and thirteen astigmatic participants with substandard corrected visual acuity (VA; defined as meridional amblyopia, MA group, 19 eyes, 7 males, mean age = 13.08 ± 6.75 years) were also recruited in the current study. Their refractive profiles were summarized in Table 1, and clinical demographics details were provided in Supplementary Table 1. Five amblyopes entered this study within a few years (3.89 ± 1.79) of completing conventional amblyopia therapy (i.e., patching therapy). Individuals with regular astigmatism were recruited under the inclusion criteria (Chuck et al., 2018) for eyes in the AST and MA groups. Participants in the study went through careful ocular health examination, VA, autorefraction, manifest subjective refraction, motility examination (including cover-uncover and alternate-cover testing), near stereopsis (marked as Vision Assessment Cooperation^TM^ V01, United States), distance stereopsis (Stereo Optical Distance Randot^®^ Stereotest, United States), and corneal topography assessments. Cases of strabismus or micro-strabismus, ocular diseases, and/or abnormalities were excluded.

**TABLE 1:** Summary of the refractive profile of MA and AST groups in this study showing the mean refractive error (in DS and DC), power range (in DS and DC), spherical equivalent (in D), the refractive and occlusion history of the participants.

	AST	MA
N	10 (20 eyes);3/10 astigmatism with anisometropia	13 (19 eyes);6/13 meridional amblyopia with anisometropia
Age	Mean 9.10 ± 1.37	Mean 13.08 ± 6.75
	median 9	median 11
	age range 8–12	age range 7–26
BCVA	OD −0.00 ± 0.04	OD 0.22 ± 0.10
	OS 0.01 ± 0.04	OS 0.16 ± 0.06
VA	OD 0.31 ± 0.19	OD 0.50 ± 0.21
	OS 0.34 ± 0.23	OS 0.41 ± 0.14
Mean refractive error (DS/DC)	OD + 1.15 DS/−3.08 DC	OD + 1.78 DS/−3.56 DC
	OS + 1.03 DS/−3.13 DC	OS + 1.66 DS/−3.30 DC
Power range (DS/DC)	OD −2.50 to + 2.50 DS/−6.00 to −1.75 DC	OD −3.75 to + 6.50 DS/−5.50 to −1.50 DC
	OS −2.75 to + 2.75 DS/−6.00 to −1.50 DC	OS −4.00 to + 5.75 DS/−6.00 to −2.00 DC
Spherical equivalent (mean ± SD)	OD 0 ± 3.51 D	OD −0.39 ± 1.41 D
	OS 0.01 ± 3.29 D	OS −0.54 ± 1.69 D
Refractive history	Current spectacle wearers	Current spectacle wearers
Occlusion history	N.A.	5/13 were treated with patching therapy (3.89 ± 1.79 years)

*N, number of participants; OD, right eye; OS, left eye; and N.A., not applicable.*

All participants completed the CSF tests, and nine participants in the AST group (13 eyes) and twelve participants in the MA group (16 eyes) completed the EEG scalings. Other participants did not complete the EEG scalings for personal reasons (e.g., limited time, low willing for receiving EEG scalings). This study followed the tenets of the Declaration of Helsinki and was approved by the Zhongshan Ophthalmic Center Ethics Committee. Informed consent was obtained from all participants (or their guardian) prior to data collection. All the participants were wearing 1 month new spectacles or contact lenses under optimal optical corrections prescribed by the same experienced optometrist (author FL) at Zhongshan Ophthalmic Center, in order to avoid the influence of new optical adaptation and previous incorrect optical fitting.

### Procedure

Stimuli were displayed on a gamma-corrected AOC G2460PQU/BR LCD computer monitor (120 Hz refresh rate, 1920 × 1080 resolution, and 53.1 cm × 29.8 cm). All experiments were controlled by a PC running MATLAB (Mathworks Inc. Natick, MA, United States) and Psychophysics Toolbox (Brainard, 1997; Pelli, 1997). A special circuit was used to produce 14-bit gray-level resolution (Li et al., 2003; Li and Lu, 2012). The mean background luminance was 27 cd/m^2^. During the whole experiment, the participant put the head on a chin rest and viewed the stimuli monocularly in a dimly lit room, with the tested eye watching while another eye occluded.

Participants went through a battery of measurements, including EDTRS VA, CSF tests along two perpendicular meridians (i.e., horizontal and vertical meridian, to match the distribution of regular astigmatism), and sVEP tests along the same two perpendicular meridians. Both the CSFs and sVEPs were measured monocularly.

### The Tasks

#### CSF Test

The CSF paradigm was applied to assess the CSF along two meridians. While measuring CSF, the display subtended 4° × 4° at a viewing distance of 1.50 m for participants. To minimize edge effects, a half-Gaussian ramp (σ = 0.5°) was used to blend the gratings into the background. We measured contrast thresholds in a two-interval forced choice grating detection task at seven spatial frequencies (0.5, 1, 2, 4, 8, 12, and 16 c/d) using a three-down one-up staircase procedure that decreased signal contrast by 10% (multiplied the previous value by 0.9) after every three consecutive correct responses and increased signal contrast by 10% after every incorrect response, converging to a performance level of 79.3% correct (Huang et al., 2008; Lu and Dosher, 2014). Following previous studies, each trial started with a 200-ms fixation cross in the center of the display. This was followed by two 200-ms intervals, signaled by a brief tone in the beginning of each and separated by 500 ms. A grating was randomly presented in one of the two intervals. The other interval was blank. Participants indicated the signal interval by pressing one of the keys (F/J keypress) with index finger. The response also initiated the next trial. A reversal results when the staircase changes from increasing to decreasing contrast or vice versa. Usually, one block of three-down one-up staircase with a 10% step size produces about a dozen reversals in 80 trials (Zhang et al., 2019). To make sure getting enough reversals, we set 100 trials to measure the contrast threshold at each spatial frequency. Following the standard practice, we averaged the contrasts of an even number of reversals to estimate the contrast threshold after excluding the first three or four reversals.

Vertical and horizontal sinusoidal gratings were adopted to measure CSF along two meridians, respectively. The seven spatial frequency conditions were randomly mixed, with two meridian blocks completed in random order under each spatial frequency condition. Thus, there were fourteen blocks with 100 trials per block. A 10 min demo program was applied to run before formal data collection to avoid learning effect. A formal testing session of about 1.5 h was required to complete the CSF test in each eye. The sequence of the CSF tests in the two eyes was randomized and counterbalanced across participants.

#### EEG Data Acquisition and sVEP Test

Participants were seated in a shielded room. The EEG signals were amplified and digitized using an Active Two 64-channel Amplifier with the 64-channel Cap in accordance to the international 10–20 system (Biosemi, Netherland), which provided fast and simple electrode placement. Signals from 64 electrodes were recorded and the impedance of each electrode was kept below 10 kV. Horizontal and vertical electrooculograms (HEOG and VEOG) were also recorded to monitor eye movements. Two reference electrodes were used. The data were sampled at 2048 Hz and filtered with a 0.16–100 Hz band-pass filter.

**FIGURE 1:**
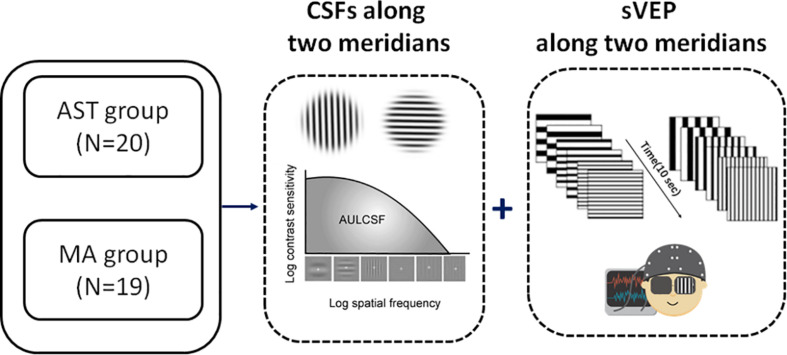
Experimental flowchart. We measured the CSF and sVEP signals along two principal meridians (i.e., horizontal meridian and vertical meridian, to match the distribution of with-the-rule astigmatic subjects in the study) monocularly in all participants. *N* indicates the number of eyes. See also Supplementary Table 1 for clinical details.

For spatial sVEP, sweeps of spatial frequency at high contrast were adopted (see Figure 1), and the parameters of stimulus refer to previous research (Hou et al., 2011). An 80% contrast, 6-Hz, phase-reversing cosine grating (shown as a square-wave grating) was swept from 2 to 16 cyc/deg in 10 linear steps. The sweep duration was 10 s. The display subtended 10° × 10° at a viewing distance of 0.90 m for participants. Spatial sVEP test was also monocularly conducted, and the non-viewing eye was occluded with a black eye patch.

### Data Analysis

#### CSF Data

The AULCSF and CSF acuity were calculated (Dorr et al., 2018). Both the spatial frequency and the contrast sensitivity in the logarithmic value were generated. We computed the area under the log CSF (AULCSF) for spatial frequencies ranging from 1.5 to 18 cpd using the trapezoid method. We also computed CSF Acuity, i.e., the intersection of the CSF with the *x* axis (where contrast threshold is 100%). Additionally, the SF threshold at 80% ctr was calculated and extracted in order to investigate the relationship between CSF and sVEP tests.

#### sVEP Data

The EEG was analyzed using a customized toolbox (mfeeg. http://sourceforge.net/p/mfeeg) programmed with MATLAB (The Mathworks, Natick, MA, United States). Continuous EEG recordings were band-pass filtered from 1 to 30 Hz.

The stimulus is presented at a given temporal frequency (6 Hz in this study) that drives visual cortical neurons at that frequency and at exact integer multiples of that frequency, as well as the stimulus is in the visible range. The visual response synchronized to the display is sampled with appropriately positioned leads, and the VEP amplitude versus stimulus intensity function is measured as the stimulus-driven response drops into the background EEG noise. In this study, swept parameter presentations were repeated ten times.

To measure the response functions, sVEP recordings for each 10-s trial were divided into 10 sequential epochs that corresponded to the swept stimulus values. For each epoch, a recursive least square (RLS) adaptive filter was used to generate a series of complex valued spectral coefficients representing the amplitude and phase of response components tuned to its multiples of the stimulus frequency (Tang and Norcia, 1995). The signals from three electrodes (Oz, O1, and O2) were averaged for further analysis and we focused on the second harmonics (12 Hz in the current study) of the temporal frequency (Almoqbel et al., 2008, 2011; Hou et al., 2018). These spectral coefficients for each epoch were coherently averaged across trials for each subject and stimulus conditions (Hou et al., 2018). These functions were also used to estimate thresholds for each subject’s individual conditions.

#### sVEP Threshold Estimation

Response thresholds were estimated by regression of amplitudes from the trial-average epochs for each swept stimulus condition of each subject (Hou et al., 2018). We applied the regression procedure to the sweep response function of each individual subject. For those individuals whose response functions along two meridians both passed the regression criteria, we calculated the resultant thresholds. The regression criteria was adopted from previous research (Norcia and Tyler, 1985), which chose an SNR (signal to noise ratio) of >3:1 (i.e., the amplitude of the peak response signal has to be at least three times larger than the adjacent noise frequency) as a criterion (Norcia and Tyler, 1985).

We also calculated the amplitudes by averaging the sweep response functions of individual participants for a given stimulus condition. In this analysis, each participant contributed equally to all conditions. Error bars in the figures depicting sweep responses are vector standard errors of the vector mean (Hou et al., 2007).

### Statistical Analysis

The statistical software package SPSS (version 19; IBM Corp, Armonk, NY, United States) was used for analysis. In order to account for right and left eye-related data (Glynn and Rosner, 2012), linear mixed models (LMMs) analysis was used to investigate the effect of stimulus meridian (strong or weak meridian) and group on the outcome measures. Bonferroni correction was applied to multiple paired comparisons to correct for family-wise error. According to previous research (Huang et al., 2018), monocular data from two eyes was in the same comparison group in the current study design, making *F*-test of LMMs no better than two sample *t*-test. Moreover, *t*-test using the average of two eyes would not perform best considering the small sample size. Thus, comparisons between two meridians were reported with two sample *t*-test.

## Results

### CSF Results

**FIGURE 2:**
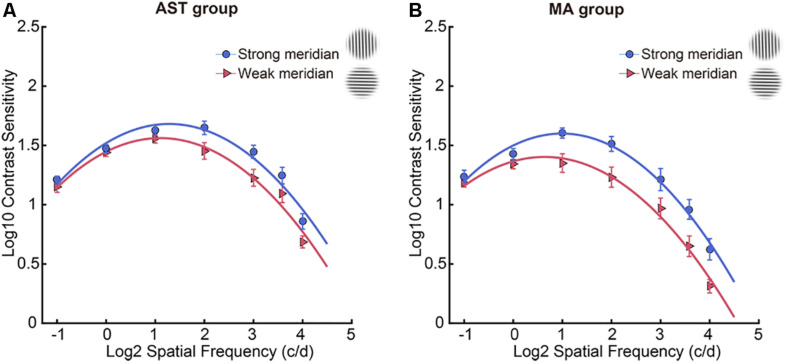
CSF results along two meridians. Participants showed meridional disparity in the AST group **(A)** and MA group **(B)**. Error bars stand for ± S.E.M.

Contrast sensitivity function results along two meridians were shown in Table 2, and meridional disparity was present in the AST group (Figure 2A) and MA group (Figure 2B). Individual CSF result for each participant was provided in Supplementary Figure 1. LMMs analysis was used to investigate the effect of stimulus meridian (strong or weak meridian) and group on the AULCSF and Cutoff acuity.

**TABLE 2:** Mean values of sVEP threshold, amplitudes at target frequency (12 Hz), and CSF tests along two meridians.

	Strong meridian	Weak meridian	Difference	*t*-value	*p*-value
	(Mean ± SE)	(Mean ± SE)	(Mean ± SE)		
**sVEP results at 12 Hz**					
**AST group**					
Threshold (*N* = 13)	19.201 ± 0.675	16.621 ± 0.569	2.580 ± 0.881	2.928	0.013*
sf1(2 cpd)	4.235 ± 0.362	3.580 ± 0.500	0.292 ± 0.814	0.359	0.729
sf2(3.6 cpd)	3.539 ± 0.288	3.274 ± 0.254	0.362 ± 0.190	1.907	0.073
sf3(5.1 cpd)	4.211 ± 0.379	2.997 ± 0.273	1.214 ± 0.258	4.713	< 0.001**
sf4(6.7 cpd)	4.096 ± 0.391	2.909 ± 0.244	1.265 ± 0.298	4.240	< 0.001**
sf5(8.2 cpd)	3.857 ± 0.394	2.898 ± 0.192	0.921 ± 0.372	2.475	0.024*
sf6(9.8 cpd)	3.276 ± 0.307	2.303 ± 0.192	1.051 ± 0.298	3.529	0.002*
sf7(11.3 cpd)	2.909 ± 0.258	2.385 ± 0.164	0.586 ± 0.309	1.896	0.076
sf8(12.9 cpd)	2.304 ± 0.183	2.269 ± 0.172	0.092 ± 0.231	0.396	0.697
sf9(14.4 cpd)	2.214 ± 0.201	2.230 ± 0.124	0.096 ± 0.181	0.529	0.605
sf10(16 cpd)	2.080 ± 0.111	2.262 ± 0.197	−0.272 ± 0.224	–1.216	0.243
**MA group**					
Threshold (*N* = 16)	17.113 ± 0.634	14.803 ± 0.529	2.310 ± 0.610	3.789	0.002*
sf1(2 cpd)	3.997 ± 0.432	4.558 ± 0.356	−1.453 ± 0.512	–2.835	0.025*
sf2(3.6 cpd)	3.524 ± 0.293	3.018 ± 0.149	0.506 ± 0.292	1.735	0.100
sf3(5.1 cpd)	3.834 ± 0.379	2.911 ± 0.193	0.923 ± 0.362	2.552	0.020*
sf4(6.7 cpd)	3.859 ± 0.338	2.655 ± 0.193	1.204 ± 0.293	4.111	0.001*
sf5(8.2 cpd)	3.480 ± 0.352	2.376 ± 0.204	1.104 ± 0.289	3.826	0.001*
sf6(9.8 cpd)	3.123 ± 0.383	2.149 ± 0.198	1.050 ± 0.376	2.791	0.013*
sf7(11.3 cpd)	2.823 ± 0.388	2.082 ± 0.163	0.741 ± 0.378	1.959	0.066
sf8(12.9 cpd)	2.439 ± 0.253	1.968 ± 0.173	0.450 ± 0.231	1.951	0.068
sf9(14.4 cpd)	2.024 ± 0.207	1.937 ± 0.176	0.103 ± 0.243	0.422	0.679
sf10(16 cpd)	2.360 ± 0.207	1.977 ± 0.173	0.256 ± 0.218	1.176	0.261

**CSF measures**					

**AST group**					
SF threshold	41.919 ± 2.786	34.681 ± 2.114	7.238 ± 2.829	2.558	0.019*
AULCSF	1.574 ± 0.050	1.411 ± 0.054	0.163 ± 0.032	5.030	< 0.001**
CSF acuity	1.642 ± 0.028	1.565 ± 0.026	0.077 ± 0.033	2.362	0.029*
0.5 cpd	1.212 ± 0.029	1.153 ± 0.048	0.059 ± 0.038	1.563	0.135
1 cpd	1.473 ± 0.033	1.443 ± 0.035	0.031 ± 0.025	1.212	0.240
2 cpd	1.626 ± 0.043	1.558 ± 0.038	0.068 ± 0.040	1.718	0.102
4 cpd	1.648 ± 0.057	1.454 ± 0.070	0.194 ± 0.056	3.464	0.003*
8 cpd	1.446 ± 0.056	1.227 ± 0.071	0.219 ± 0.049	4.444	< 0.001**
12 cpd	1.245 ± 0.070	1.097 ± 0.079	0.149 ± 0.065	2.287	0.034*
16 cpd	0.859 ± 0.065	0.686 ± 0.050	0.173 ± 0.065	2.667	0.015*
**MA group**					
SF threshold	32.107 ± 3.014	21.949 ± 1.478	10.158 ± 2.143	4.740	< 0.001**
AULCSF	1.397 ± 0.066	1.113 ± 0.077	0.284 ± 0.028	10.209	< 0.001**
CSF acuity	1.508 ± 0.042	1.370 ± 0.026	0.138 ± 0.029	4.707	< 0.001**
0.5 cpd	1.238 ± 0.053	1.185 ± 0.036	0.052 ± 0.037	1.400	0.178
1 cpd	1.426 ± 0.047	1.343 ± 0.041	0.083 ± 0.033	2.554	0.020
2 cpd	1.604 ± 0.042	1.351 ± 0.078	0.253 ± 0.050	5.060	< 0.001**
4 cpd	1.512 ± 0.063	1.232 ± 0.085	0.280 ± 0.043	6.505	< 0.001**
8 cpd	1.212 ± 0.093	0.968 ± 0.087	0.243 ± 0.052	4.717	< 0.001**
12 cpd	0.960 ± 0.083	0.650 ± 0.087	0.310 ± 0.058	5.337	< 0.001**
16 cpd	0.624 ± 0.090	0.313 ± 0.057	0.311 ± 0.045	6.946	< 0.001**

*Post hoc* comparisons between strong meridian and weak meridian are also presented. A single asterisk * indicates a significance level of *p* < 0.05. Two asterisks ** indicate a significance level of *p* < 0.001.

**FIGURE 3:**
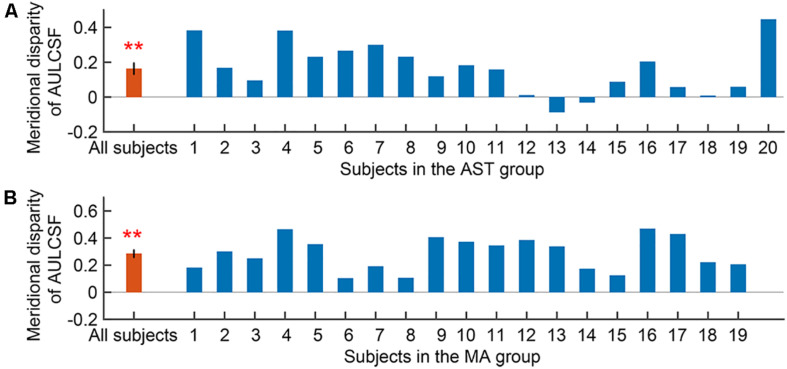
Meridional disparity of AULCSF in the AST group **(A)** and the MA group **(B)**. Error bars represent ± S.E.M. Two asterisks ** indicate a significance level of *p* < 0.001.

We first evaluated the meridional disparity of AULCSF. LMMs analysis showed a significant effect of meridian (*F*_1,52.44_ = 46.812, *p* < 0.001), a marginal significant main effect of group (*F*_1,20.22_ = 3.176, *p* = 0.090), and a marginal significant interaction between the two factors (*F*_1,52.44_ = 3.461, *p* = 0.070). *F*-test of LMMs based on estimated marginal means indicated a significant meridional disparity of AULCSF (*M*_*diff*_ = 0.223 ± 0.033, *F*_1,52.44_ = 46.812, and *p* < 0.001). Significant meridional disparity of AULCSF was found in the AST (*M*_*diff*_ = 0.163 ± 0.032, *t*_19_ = 5.030, and *p* < 0.001; Figure 3A) and MA (*M*_*diff*_ = 0.284 ± 0.028, *t*_18_ = 10.209, and *p* < 0.001; Figure 3B) groups. In addition, the MA group showed larger meridional disparity of AULCSF compared to the AST group (*t*_37_ = 2.834, *p* = 0.007).

**FIGURE 4:**
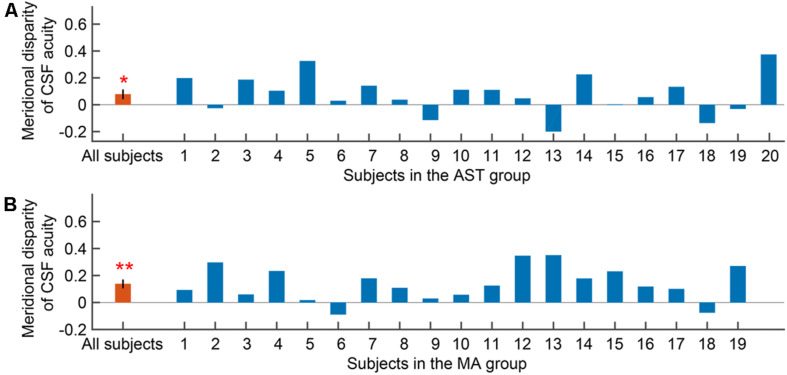
Meridional disparity of CSF acuity in the AST group **(A)** and the MA group **(B)**. Error bars represent ± S.E.M. An asterisk * indicates a significance level of *p* < 0.05. Two asterisks ** indicate a significance level of *p* < 0.001.

We then evaluated the meridional disparity of CSF acuity. LMMs analysis showed a significant effect of meridian (*F*_1,51.33_ = 18.142, *p* < 0.001), and group (*F*_1,18.58_ = 10.793, *p* = 0.004). The interaction between the two factors was not significant (*F*_1,51.33_ = 1.425, *p* = 0.238). *F*-test of LMMs based on estimated marginal means indicated a significant meridional disparity of CSF acuity (*M*_*diff*_ = 0.107 ± 0.025, *F*_1,51.33_ = 18.142, and *p* < 0.001). Significant meridional disparity of CSF acuity was found in the AST (*M_*diff*_* = 0.077 ± 0.033, *t*_19_ = 2.362, and *p* = 0.029; Figure 4A) and MA group (*M*_*diff*_ = 0.138 ± 0.029, *t*_18_ = 4.707, and *p* < 0.001; Figure 4B). Meanwhile, there was no significant difference between meridional disparity of CSF acuity in two groups (*t*_37_ = 1.367, *p* = 0.180).

Furthermore, we evaluated the meridional disparity of contrast sensitivity corresponding to individual SF. LMMs analysis showed a significant main effect of meridian (*F*_1,496.69_ = 90.249, *p* < 0.001), a significant main effect of SF (*F*_6,496.69_ = 174.645, *p* < 0.001), a marginal significant main effect of group (*F*_1,20.57_ = 3.053, *p* = 0.096), a significant interaction effect between meridian and group (*F*_1,496.69_ = 6.292, *p* = 0.012), a significant interaction effect between SF and group (*F*_6,496.69_ = 8.155, *p* < 0.001), a significant interaction effect between meridian and SF (*F*_6,496.69_ = 3.060, *p* = 0.006), and but a non-significant interaction between the three factors (*F*_6,496.69_ = 0.563, *p* = 0.759). *F*-test of LMMs further showed the existence of meridional disparity of contrast sensitivity corresponding to several spatial frequencies (2, 4, 8, 12, and 16 c/d), *F*_1,496.69_ = 11.069, *p* = 0.001; *F*_1,496.69_ = 24.160, *p* < 0.001; *F*_1,496.69_ = 22.923, *p* < 0.001; *F*_1,496.69_ = 22.604, *p* < 0.001; and *F*_1,496.69_ = 25.119, *p* < 0.001, respectively. *Post hoc* comparisons between two meridians are listed in Table 2.

### sVEP Results

For sVEP data, we first evaluated the meridional disparity of sVEP thresholds (Table 2). LMMs analysis showed a significant effect of meridian (*F*_1,54_ = 16.234, *p* < 0.001), and group (*F*_1,54_ = 10.354, *p* = 0.002). The interaction between the two factors was not significant (*F*_1,54_ = 0.050, *p* = 0.825). *F*-test of LMMs based on estimated marginal means indicated a significant meridional disparity of sVEP thresholds (*M*_*diff*_ = 2.445 ± 0.607, *F*_1,54_ = 16.234, and *p* < 0.001). Significant meridional disparity of sVEP threshold was found in the AST (*M*_*diff*_ = 2.580 ± 0.881, *t*_12_ = 2.928, and *p* = 0.013) and MA (*M*_*diff*_ = 2.310 ± 0.610, *t*_15_ = 3.789, and *p* = 0.002) groups. Meanwhile, the meridional disparity of sVEP threshold in the MA group was no larger than that in the AST group (*t*_27_ = 0.259, *p* = 0.798).

**FIGURE 5:**
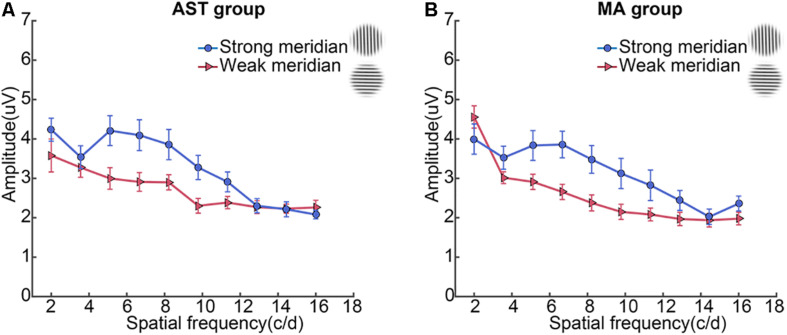
Spatial sVEP voltage response at the second harmonic of the test stimulus reversal rate is plotted as a function of spatial frequency of the test stimulus in the AST group **(A)** and the MA group **(B)**. Error bars represent ± S.E.M.

We then examined the meridional disparity of amplitudes for spatial frequencies presented (Figure 5; also see Table 2). LMMs analysis showed a significant main effect of meridian (*F*_1,665.08_ = 64.807, *p* < 0.001), a significant main effect of SF (*F*_9,665.05_ = 34.494, *p* < 0.001), a significant interaction effect between meridian and SF (*F*_9,665.12_ = 4.959, *p* < 0.001, but a non-significant main effect of group (*F*_1,20.75_ = 0.241, *p* = 0.628). No significant interaction between meridian and group (*F*_1,665.08_ = 0.043, *p* = 0.835), between SF and group (*F*_9,665.05_ = 1.025, *p* = 0.418), or between the three factors (*F*_9,665.12_ = 0.950, *p* = 0.481) was found. Multiple comparison analysis further showed the existence of meridional disparity of amplitudes corresponding to medium SF (5.1, 6.7, 8.2, 9.8, and 11.3 c/d), *F*_1,664.87_ = 25.053, *p* < 0.001; *F*_1,664.89_ = 32.381, *p* < 0.001; *F*_1,664.89_ = 23.397, *p* < 0.001; *F*_1,665.01_ = 22.570, *p* < 0.001; and *F*_1,664.95_ = 10.252, *p* = 0.001, respectively. *Post hoc* comparisons between two meridians are listed in Table 2.

### Correlation Between CSF and sVEP Tests

**FIGURE 6:**
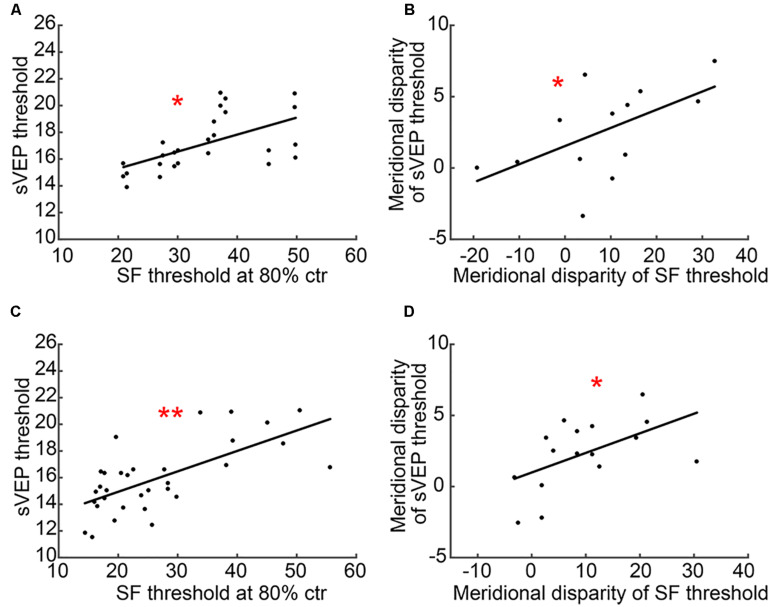
A scatter plot of CSF and sVEP measures. **(A)** SF threshold at 80% ctr and the sVEP threshold in the AST group; **(B)** meridional disparity of SF threshold at 80% ctr and meridional disparity of the sVEP threshold in the AST group; **(C)** SF threshold at 80% ctr and the sVEP threshold in the MA group; and **(D)** meridional disparity of SF threshold at 80% ctr and meridional disparity of the sVEP threshold in the MA group. An asterisk * indicates a significance level of *p* < 0.05. Two asterisks ** indicate a significance level of *p* < 0.001.

Most interestingly, we found measurable correlations between CSF (SF threshold at 80% ctr) and sVEP (sVEP threshold) tests. Results showed that there was a significant correlation between SF threshold at 80% ctr and the sVEP threshold in the AST (*r* = 0.578, *p* = 0.002; Figure 6A) and MA (*r* = 0.671, *p* < 0.001; Figure 6C) groups. Moreover, the meridional disparity of SF threshold at 80% ctr and meridional disparity of the sVEP threshold was also significantly correlated in the AST (*r* = 0.569, *p* = 0.043; Figure 6B) and MA (*r* = 0.532, *p* = 0.034; Figure 6D) groups.

### Correlation Between Cylindrical Refractive Errors and Meridional Disparities of CSF or sVEP Measures

In a final analysis, we examined the relationship between cylindrical refractive errors and meridional disparities of CSF or sVEP measures. There was no significant correlation between cylindrical refractive errors and meridional disparities of any CSF or sVEP measure (AULCSF, CSF acuity, SF threshold at 80% ctr, and sVEP threshold; all *p* > 0.050). Consistent with previous study (Yap et al., 2020a), this indicated that cylindrical refractive error alone does not constitute the decisive factor for the level of meridional disparity, and other factors may also be related to the asymmetric development in the visual system.

## Discussion

The current study investigated the asymmetric development of spatial vision related to astigmatism in the human visual system via combining psycho-physical and EEG scalings. We assessed the CSF and sVEP along two principal meridians in participants with astigmatism when being optimally corrected with spectacles, and confirmed the horizontal and vertical asymmetry of spatial vision in human astigmatism. This finding was in line with animal studies (Blakemore and Cooper, 1970; Hirsch and Spinelli, 1970) and other human studies (Freeman et al., 1972; Fiorentini and Maffei, 1973; Mitchell et al., 1973; Freeman, 1975), suggesting that the meridian-specific partial deprivation in early life can lead to monocularly asymmetric development of spatial vision in the visual system.

Neurons in the primary visual cortex, responsive to the retina mapping projection from optical input, is highly sensitive to visual experience during the critical period (Wiesel, 1963; Morishita and Hensch, 2008). Astigmatism without appropriate optical corrections before the critical period would permanently modify the visual system and result in monocularly and binocularly abnormal visual perception (Freeman et al., 1972). It has been reported that abnormal visual input in early life would affect the establishment of visual perception, and may even lead to MA (Hubel and Wiesel, 1962; Shapley et al., 2003). In this study, converging evidence from CSF and sVEP results suggested that meridional disparity on participants with astigmatism are of neural, rather than optical, origin. In addition, we found that the meridional disparity of AULCSF was more remarkable in MA group. As the AULCSF is a summary measure of spatial vision (Applegate et al., 1998; Oshika et al., 2006; Lesmes et al., 2010), we speculated that this finding indicated a higher level of abnormal spatial vision in meridional amblyopes than astigmats, and that the phenomenon that astigmats had normal corrected VA but showed meridional disparity of spatial vision might be due to the compensation of neural system. It should be noted that higher level of meridional disparity was only found in the AULCSF measurement but not in other measures (CSF acuity, SF threshold at 80% ctr, sVEP threshold, and sVEP amplitudes). Meridional amblyopes would generally be expected to have much greater magnitude of meridional disparities. We speculated that it may be due to low severity of amblyopia or the treatment history of amblyopes. All the amblyopes were already treated via wearing spectacles prior to the measuring of the VEPs, which helped to improve the visual performance (Gao et al., 2018).

The finding that the horizontal meridian was weaker than vertical meridian in the current study, may be attributed to a horizontal effect. Horizontal effect indicated that the horizontal meridian is less sensitive than the rest of meridians. This finding was consistent with previous study showing that horizontal effect existed in both astigmats and non-astigmats (Yap et al., 2019). Therefore, it should be noted that the horizontal effect might be confounding. All the participants included in the current study were with-the-rule astigmatic, which might limit the explanation. Future research could include individuals with other types of regular astigmatism and further clarify the relationship between astigmatism type and meridional disparity.

Consistent with previous study (Yap et al., 2020a), our results demonstrated that astigmatism alone is not the decisive factor on the magnitude of their meridional disparity. We speculated that meridional disparity of spatial vision might result not only from meridional optical blur alone, but also from many alternative mechanisms, such as neural suppression of amblyopia if existed (Hess et al., 2014). Meanwhile, there exists differences between the current study and Yap’s study. They recruited newly diagnosed amblyopic children who have never worn spectacles, and this current study investigated subjects who have worn spectacles for at least a few months prior to testing. Considering that the duration of astigmatic blur (Keech and Kutschke, 1995) and treatment history (Gao et al., 2018) also influenced the magnitude of meridional disparity, the finding that meridional amblyopes demonstrated the horizontal effect in the current study might suggest a consequence of treatment or recovery. Besides, the horizontal effect was only observed in children (3–7 years old) in Yap’s study, and the meridional disparity was observed in subjects of older age range (7–26 years old) in our study. According to recent studies (Yap et al., 2019, 2020b; Yap and Boon, 2020), the horizontal effect is an indicator of normality. It is important to separate the meridian-specific deficit from the normal physiological phenomenon (i.e., horizontal effect), thus future study could include newly diagnosed amblyopes and meridional amblyopes with other types of regular astigmatism to further clarify it.

For the combination of psycho-physical methods and EEG scalings, the spatial sVEP was adopted, instead of contrast sVEP which measured contrast sensitivity, causing the mismatch of two paradigms. We adopted seven spatial frequencies in the CSF test and ten spatial frequencies in the sVEP test, which also caused the mismatching of grating stimuli in two paradigms. Even though the SF thresholds at 80% ctr and the sVEP thresholds were significantly correlated, it is not applicable to explore the relationship between contrast sensitivity and sVEP amplitude of gratings on each spatial frequency. CSF and sVEP tests with the same set of grating stimuli are expected in future research. Though we set an SNR of >3:1 as a criterion to control the overestimation following previous research (Norcia and Tyler, 1985), it is acknowledged that sweep VEPs might overestimate spatial resolution thresholds (Hamilton et al., 2020).

In the current study design, monocular data from two eyes was included in the same group. A larger sample size and data from randomly selected eye may help to clarify these observations. Other limitations of the study include: (1) the age range (7–26 years old) was relatively wide; (2) some amblyopes had VA reduction in both eyes without an interocular acuity difference significantly; (3) several amblyopes with anisometropia were not specifically excluded from this present study, which may contribute to an alternative neural mechanism of amblyopia, such as neural suppression (Hess et al., 2014); and (4) several amblyopes received occlusion treatment for years, which may already compensate for the magnitude of their meridional disparity.

Individuals with astigmatism are the optimal and natural models for exploring the asymmetric development of spatial vision, and further investigations are expected based on our current findings. Firstly, we could carry out more explorations to investigate the occurrence and development of astigmatism and MA. For instance, we could measure CSF in the real-world scenarios, or apply other neuroimaging tools, such as functional magnetic resonance imaging (fMRI) and functional near infrared spectroscopy (fNIRS). Secondly, we could reduce and even eliminate the visual deficit in the primary visual cortex by adopting perceptual learning or other vision-related interventions. Furthermore, we expect individuals with astigmatism to fully restore visual functions even without optical corrections by taking advantage of visual neural plasticity.

## Data Availability Statement

The data generated during the current study is available from the corresponding author on reasonable request.

## Ethics Statement

The studies involving human participants were reviewed and approved by Zhongshan Ophthalmic Center Ethics Committee. Written informed consent to participate in this study was provided by the participants’ legal guardian/next of kin.

## Author Contributions

JY, JL, and LG designed the research. LG, YW, and LF performed the research. LG analyzed the data and drafted the manuscript. JY, JL, and Z-LL revised the manuscript. All authors commented on and edited the manuscript, and approved the final version of the manuscript.

## Conflict of Interest

The authors declare that the research was conducted in the absence of any commercial or financial relationships that could be construed as a potential conflict of interest.
