# Supplementary material for: Meridian-Specific and Post-Optical Deficits of Spatial Vision in Human Astigmatism: Evidences From Psycho-Physical and EEG Scalings
Source: Front Psychol. 2021 Mar 17;12:595536. doi: 10.3389/fpsyg.2021.595536 (PMC8010696; doi:10.3389/fpsyg.2021.595536)
Supplement: Supplementary file 1 [file Table_1.docx]

**Supplementary materials**

| **NO.** | **Name** | **Gender** | **Age** | **OD** | **OS** | **BCVA**  **OD/OS** | **VA**  **OD/OS** | **Refraction-Error OD/OS** | **EEG scaling** | **Patching history(y)** |
| --- | --- | --- | --- | --- | --- | --- | --- | --- | --- | --- |
| **1** | TZR | M | 8 | AST | AST | 0/  0 | 0.38/  0.34 | +1.75/-3.50*180  +2.00/-4.00*175 | Y | N |
| **2** | XXQC | F | 10 | MA | MA | 0.18/  0.12 | 0.48/  0.58 | -0.75/-2.25*180  -0.25/-3.25*180 | N | N |
| **3** | YZH | M | 7 | MA | MA | 0.2/  0.18 | 0.48/  0.46 | +5.00/-2.50*5  +5.50/-2.50*160 | Y | N |
| **4** | DHB | M | 14 | MA | MA | 0.1/  0.1 | 0.32/  0.36 | +1.50/-5.50*180  +1.75/-6.00*180 | Y | Y/5.5 |
| **5** | PZX | M | 8 | AST | AST | 0/  0 | 0.12/  0.18 | +1.00/-1.75*180  +1.50/-2.25*10 | N | N |
| **6** | ZDY | M | 8 | None | MA | 0.04/  0.24 | 0.5/  0.44 | +3.75/-2.75*175  +4.25/-2.75*170 | Y | Y/4.6 |
| **7** | WJ | F | 11 | MA | None | 0.3/  0.3 | 0.6/  0.6 | +0.75/-5.50*175  -1.50/-1.00*180 | Y | N |
| **8** | LWX | F | 7 | None | MA | 0.02/  0.14 | 0.2/  0.24 | +3.75/-2.50*10  +3.50/-3.75*180 | Y | N |
| **9** | FRH | M | 11 | None | MA | 0.22/  0.1 | 0.44/  0.32 | -0.75/-1.00*175  0/-2.00*170 | Y | Y/5.4 |
| **10** | HCB | M | 12 | AST | AST | -0.08/  0.04 | 0.34/  0.16 | +1.25/-2.00*5  +1.50/-3.00*170 | N | N |
| **11** | LEL | F | 8 | AST | AST | 0/  0 | 0.1/  0.1 | +1.25/-2.75*180  +1.25/-1.75*180 | Y | N |
| **12** | LYJ | F | 9 | AST | AST | 0.04/  0.02 | 0.62/  0.6 | -2.50/-3.50*170  -2.75/-3.25*180 | Y | N |
| **13** | CRQ | M | 8 | AST | AST | 0.02/  0.08 | 0.42/  0.46 | +2.00/-2.75*180  +2.00/-3.50*175 | Y | N |
| **14** | QYZ | F | 9 | AST | AST | 0.04/  0.04 | 0.58/  0.74 | +2.50/-6.00*180  +2.50/-6.00*172 | Y | N |
| **15** | ZY | F | 11 | None | MA | 0/  0.18 | 0.24/  0.24 | +2.50/-2.50*175  +3.00/-3.00*180 | Y | N |
| **16** | LHW | M | 8 | None | MA | 0.02/  0.12 | 0.3/  0.66 | +1.75/-3.00*5  +2.00/-6.00*175 | Y | Y/2.25 |
| **17** | LXY | F | 10 | MA | MA | 0.2/  0.28 | 0.3/  0.44 | +3.00/-1.50*165  -4.00/-2.25*10 | Y | Y/1.7 |
| **18** | WF | M | 22 | MA | MA | 0.38/  0.12 | 0.56/  0.5 | +2.00/-4.75*70  -3.25/-2.75*100 | Y | N |
| **19** | ZXF | M | 9 | AST | AST | 0/  0 | 0.12/  0.1 | +1.25/-2.50*180  +0.50/-1.50*175 | Y | N |
| **20** | YZQ | F | 11 | AST | AST | -0.06/  -0.06 | 0.34/  0.54 | +0.75/-2.50*175  -1.00/-3.25*5 | N | N |
| **21** | KAR | M | 9 | AST | AST | 0.02/  0 | 0.12/  0.22 | +2.25/-3.50*5  +2.75/-2.75*170 | Y | N |
| **22** | ZYM | F | 26 | MA | None | 0.08/  -0.08 | 0.94/  0.6 | -3.75/-3.75*18  -3.25/ | N | N |
| **23** | ZXN | M | 25 | MA | MA | 0.30/  0.20 | 0.34/  0.22 | +6.50/-2.75*95  +5.75/-2.00*80 | Y | N |

***Table S1. Clinical details of the participants.*** *Both BCVA and VA were reported in the form of LogMAR. Note: M: male, F: female, OD: right eye, OS: left eye. AST:* *astigmatism without amblyopia, MA: meridional amblyopia. None: not accordant with the inclusion criteria. Y: Yes, N: No.*


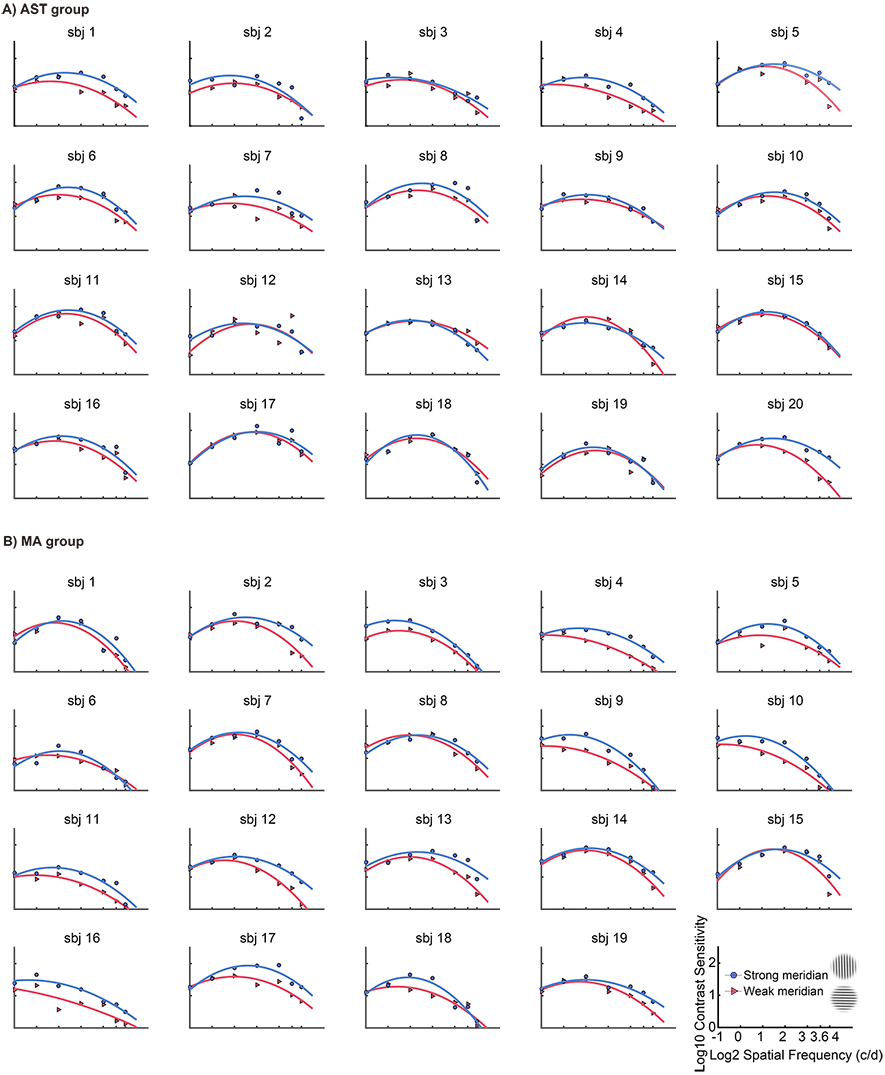


***Figure S1. Individual CSF result for participants in the AST group (A) and MA group (B).***
